# How to improve drug dosing for patients with renal impairment in primary care - a cluster-randomized controlled trial

**DOI:** 10.1186/1471-2296-13-91

**Published:** 2012-09-06

**Authors:** Antje Erler, Martin Beyer, Juliana J Petersen, Kristina Saal, Thomas Rath, Justine Rochon, Walter E Haefeli, Ferdinand M Gerlach

**Affiliations:** 1 Institute of General Practice, Goethe-University Frankfurt, Theodor-Stern-Kai 7, 60590, Frankfurt am Main, Germany; 2 Department of Internal Medicine St. Franziskus-Hospital, Hohenzollernring 72, 48145, Muenster, Germany; 3 Institute of Medical Biometry and Informatics, University of Heidelberg, Im Neuenheimer Feld 305, 69120, Heidelberg, Germany; 4 Department of Clinical Pharmacology and Pharmacoepidemiology, University Hospital Heidelberg, Im Neuenheimer Feld 410, 69120, Heidelberg, Germany

## Abstract

**Background:**

Patients with chronic kidney disease (CKD) are at increased risk for inappropriate or potentially harmful prescribing. The aim of this study was to examine whether a multifaceted intervention including the use of a software programme for the estimation of creatinine clearance and recommendation of individual dosage requirements may improve correct dosage adjustment of relevant medications for patients with CKD in primary care.

**Methods:**

A cluster-randomized controlled trial was conducted between January and December 2007 in small primary care practices in Germany. Practices were randomly allocated to intervention or control groups. In each practice, we included patients with known CKD and elderly patients (≥70 years) suffering from hypertension. The practices in the intervention group received interactive training and were provided a software programme to assist with individual dose adjustment. The control group performed usual care. Data were collected at baseline and at 6 months. The outcome measures, analyzed across individual patients, included prescriptions exceeding recommended maximum daily doses, with the primary outcome being prescriptions exceeding recommended standard daily doses by more than 30%.

**Results:**

Data from 44 general practitioners and 404 patients are included. The intervention was effective in reducing prescriptions exceeding the maximum daily dose per patients, with a trend in reducing prescriptions exceeding the standard daily dose by more than 30%.

**Conclusions:**

A multifaceted intervention including the use of a software program effectively reduced inappropriately high doses of renally excreted medications in patients with CKD in the setting of small primary care practices.

**Trial registration:**

Current Controlled Trials ISRCTN02900734

## Background

Chronic kidney disease (CKD) is a common and costly condition. In the United States, the estimated prevalence for chronic renal impairment in adults is 13% [1,2], and a similar figure is assumed for Germany. 6.4% of Medicare health expenditures in the U.S. are spent for CKD patients [3]. Major risk groups are patients with hypertension, diabetes, obesity, and dyslipidemia [4,5]. Prevalence of CKD rises exponentially in the elderly [6], and has been shown to be as high as 48% in patients over 70 years [7]. This is attributed to an age-related loss of nephron function [8] and a higher prevalence of predisposing risk factors (e.g. hypertension, diabetes) in older adults [7]. Other comorbid conditions common in elderly populations and associated with CKD are coronary artery disease, peripheral vascular and cerebrovascular disease with cognitive impairment. Comorbidities such as prostate hypertrophy and congestive heart failure result in a higher risk for acute kidney injury and kidney failure [7]. Conversely, CKD is an independent risk factor for cardiovascular disease and all-cause mortality [9].

In an aging population the incidence of CKD will likely continue to rise [10]. Early detection, appropriate treatment of risk factors, and timely referral to a nephrologist have the potential to slow CKD progression, decrease morbidity and mortality from cardiovascular disease, and lower costs [4,11]. The care family physicians provide to populations at risk puts them in a position to help detect CKD early and prevent progression [12]. Detection of CKD requires estimation of creatinine clearance, because the measurement of serum creatinine alone can be misleading, especially in elderly populations [13,14]. However, a study found that 86% of family physicians only took serum creatinine concentration into account when prescribing drugs for elderly patients [8]. If impaired renal function is detected, patients may require a dosage reduction of renally excreted drugs to avoid accumulation and adverse drug reactions [15-17]. However, recommendations on dosage adjustment are missing in up to 70% of the summaries of product characteristics provided by the pharmaceutical industry, and in other information sources available to family physicians [18], leading to the prescription of inappropriately high medication dosages in primary care [19].

Most interventions shown to be effective in improving correct dosage adjustment of medications in CKD patients involved inpatient settings, such as concurrent feedback by pharmacists [20,21], or the integration of a decision support system (CDSS) into a computerized physician order entry (CPOE) system [22,23]. However, these interventions appear less feasible in small, isolated primary care settings where personnel and financial resources are limited [24].

Similar to Germany, 26% of primary care practices in the United States are solo or 2-partner practices, and 22% are located in rural areas with limited access to clinical pharmacists [25]. 46% of American and 72% of German family physicians have an electronic health record (EHR), but the availability of decision support is limited [26]. In order to take advantage of their prime position to detect and manage patients with impaired renal function, family physicians need simple and inexpensive tools to estimate glomerular filtration rate, adjust medications correctly, and educate patients under the time and financial constraints of daily practice. Therefore, the objective of this study was to evaluate whether a primary care-based multifaceted intervention, including interactive training for family physicians and the use of a software programme for the estimation of renal function and corresponding dosage adjustment, might be effective in reducing inappropriate prescriptions in patients with impaired renal function.

## Methods

### Ethics statement

The institutional review board of the Goethe University Frankfurt approved the study protocol on September 18, 2006. All participants provided written informed consent.

### Setting and participants

All 1,876 family physicians registered with the Association of Statutory Health Insurance Physicians in South Hesse, Germany (mandatory registration) were mailed a letter informing them of the trial and inviting them to participate in an informational meeting. As the registration list contained only names and addresses, we ascertained eligibility during the informational meetings or when a physician contacted the study team directly. We stopped recruiting when the required sample was enrolled, even though more practices were interested in participating. This pragmatic recruitment procedure has been adopted in other cluster-randomized controlled trials in German primary care settings (e.g. [27]) Inclusion criteria for practices were registration as a family physician without specialization and availability of a computer in the consulting room. We excluded practices which already used a software programme for dosage adjustment in CKD.

We included patients with an estimated glomerular filtration rate (GFR) <50 ml/min and a diagnosis of chronic renal impairment (ICD-10 code N18.- or N19.-), as well as elderly patients (≥70 years) suffering from hypertension (ICD-10 code I10.-), thus defining an important risk group. Exclusion criteria were end-stage renal disease requiring dialysis, palliative care, and persons who were not regular patients of the study practice.

### Randomization and intervention

Practices identified eligible patients and sent a list of patient pseudonyms to the Institute of General Practice at Goethe-University Frankfurt (IfA). From this list, 10 patients per practice were selected by the study team using random numbers produced by SPSS (Version 15.0, SPSS Sciences, Chicago, USA). The same method was employed to allocate practices to intervention and control group in a 1:1 ratio without further stratification. Blinding practices to their group allocation was not feasible because of the nature of the intervention.

The multifaceted intervention consisted of 1) an interactive 1-hour workshop for physicians on detection and management of CKD, 2) provision of a desktop checklist of medications to be reduced or avoided in patients with CKD, 3) provision of patient information leaflets, and 4) training in the use of the software “DOSING” (accessible at http://www.dosing.de). The software DOSING contains an information database on fractional renal excretion of currently >800 compounds, allows quick and easy estimation of a patient’s creatinine clearance, and subsequently estimates individual dosage requirements. Depending on the extent of renal impairment, recommendations may be to avoid a drug (contraindication) or to reduce the dose on the basis of *Dettli*’s rules to avoid excessive exposure and potential toxicity [16,28]. In the case of metformin, the programme allowed calculation of an individual dose, but also noted that current drug labels regard the use of metformin as inappropriate in patients with renal impairment.

The software is continuously updated by the Department of Clinical Pharmacology and Pharmacoepidemiology, University Hospital Heidelberg, Germany.

Linking the DOSING software directly to the study practices’ electronic health records (EHR) was not feasible, because of the many different practice software systems in use. Therefore, the intervention practices received a stand-alone CD version, and the physicians were required to manually enter data to calculate creatinine clearance and dose modifications. During the intervention period, they received monthly telephone reminders to use the programme.

Family physicians in the control group did not receive any training or materials on CKD and were advised to continue to treat their patients as before (usual care). All written material used in this study was translated into English and is available from the authors on request.

### Outcomes

The primary and secondary outcomes evaluated at six months were the number of patients receiving at least one prescription exceeding 1) the recommended *standard* daily dosage by more than 30% and 2) the recommended *maximum* daily dose. Dose requirements for a patient were calculated as previously described [20]. Briefly, the software programme DOSING calculated a percentage by which the standard daily or maximum daily dose given to a patient with normal renal function should be reduced on the basis of the patient’s estimated creatinine clearance. We chose these outcomes rather than the number of prescriptions exceeding the recommended dosage because one overdosed drug is sufficient to potentially harm the patient.

Further secondary outcomes were the number of patients with prescriptions that are potentially dangerous or contraindicated in CKD patients such as metformin, nitrofurantoin, or allopurinol.

### Data collection

Family physicians collected data from case report forms at baseline and at 6 months. The forms were pilot-tested and revised by three of our teaching practices. They contained questions on patients’ demographic and clinical data, such as present medication, weight, and serum creatinine. All identifying patient data from the study practices was pseudonymized, i.e. only the study practice was able to identify patients. For quality assurance, the data was entered twice into a Microsoft Access database version 2000 (Microsoft Corp., Redmond, Washington, USA).

### Sample size calculation

Sample size was calculated on the basis of the primary outcome. Using patients as the unit of analysis, we estimated that at least 366 patients (183 per group) were required to detect a 15% reduction (from 50% to 35% based on results from previous studies [21,23]) with a power of 1 − β = 0.80 by means of a continuity corrected *χ*^2^-test at a 0.05 two-sided significance level. Assuming an intra-cluster correlation coefficient (ICC) of 0.01 at practice level [29] and an average cluster size of 10, we estimated a design effect of D = 1 + (10–1) x 0.01 = 1.09. Thus, to have adequate power, the sample size had to be adjusted to 400 patients (200 per group) [30]. With 10 patients per practice, we needed to include 40 practices (20 per arm). To allow for 10% lost to follow-up, we increased this number to 46 practices (23 in each arm).

### Statistical analysis

Primary efficacy analysis was a comparison between intervention and control groups at six months without and with adjusting for the following variables: underlying diagnosis, gender, patient’s age, creatinine value, and proportion of patients with at least one prescription exceeding the recommended dose at baseline. Missing values for binary outcome variables were imputed with 0 (= not exceeding recommended dose) in both groups. Differences between groups were analyzed by means of generalized estimation equations (GEEs), which take clustering by practice into account. We used empirical-based standard error estimates and set the significance level to 0.05 (two-sided). We calculated odds ratios (OR) and corresponding 95% confidence intervals (CI). Statistical analyses were carried out with SAS version 9.1 (SAS Institute, Cary, USA).

## Results

**Figure 1:**
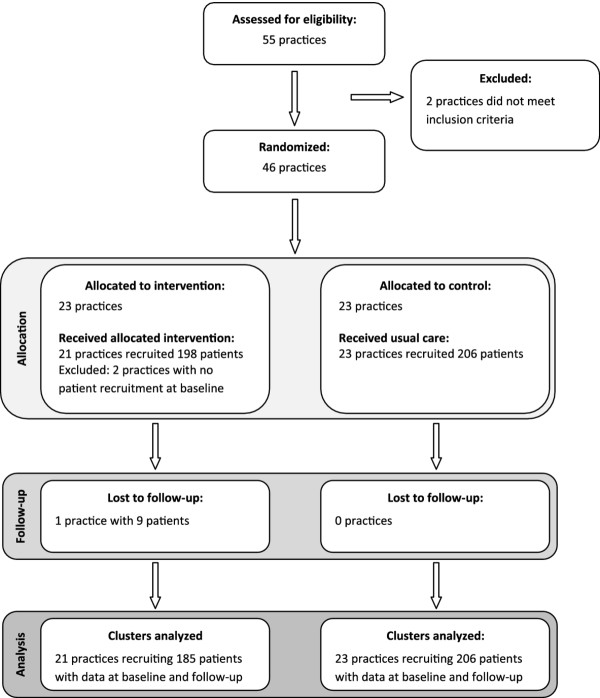
Flowchart of study practices and patients.

We assessed 55 practices for eligibility, and excluded 2 practices because they did not meet the inclusion criteria. 7 practices withdrew consent because of time constraints. 46 practices were randomized into intervention (n = 23) and control group (n = 23). 2 intervention practices dropped out without providing any baseline data and 1 intervention practice dropped out during the trial. No practice assigned to usual care crossed over to intervention or vice versa (Figure 1). We included data from 21 intervention and 23 control practices in the analysis.

The participating physicians recruited 404 study patients (198 in the intervention and 206 in the control group). 211 (52.2%) patients had a diagnosis of CKD, whereas 193 (47.8%) had a primary diagnosis of hypertension and were newly diagnosed with CKD. We collected follow-up data from 384 (95.1%) of the patients at 6 months.

### Demographic and clinical characteristics

Study practices in intervention and control group were well balanced for physicians’ age, sex, specialty (family physician or general internist), practice form and size (Table 1). The control group contained slightly more family physicians from an urban setting.

**Table 1:** Baseline characteristics of participating family physicians by intervention and control group

**Variable**	**Intervention group**	**Control group**
Total N	21	23
Age [years], mean (SD)	47.8 (7.5)	50.2 (8.1)
Male [N] (%)	11 (52.4)	16 (69.9)
Geographical location urban [N] (%)	5 (23.8)	11 (47.8)
Single-handed practice [N] (%)	12 (57.1)	14 (60.9)
Practice size (patients per quarter) mean (SD)	1238 (718)	1487 (771)
<500 patients/quarter [N] (%)	2 (9.5)	0 (0)
500–1000 patients/quarter [N] (%)	5 (23.8)	7 (30.4)
1000–1500 patients/quarter [N] (%)	8 (38.1)	5 (21.7)
>1500 patients/quarter [N] (%)	6 (28.6)	11 (47.8)

SD = standard deviation.

Patients in the two study arms were well balanced for weight, serum creatinine values, estimated creatinine clearances, mean number of prescriptions per patient, and primary diagnosis (renal impairment/hypertension). However, patients in the intervention group were slightly older and more often male (Table 2).

**Table 2:** Baseline characteristics of study patients by intervention and control group

**Variable**	**Intervention group (N = 198 patients)**	**Control group (N = 206 patients)**	**GEE model p value**
Male [N] (%)	81 (40.9)	63 (30.6)	0.033
Age [years] mean (SD)	81.4 (5.6)	79.5 (8.7)	0.005
Weight [kg] mean (SD)	70.6 (12.5)	71.8 (14.1)	0.376
Serum creatinine [mg/dl] mean (SD)	1.47 (0.44)	1.52 (0.55)	0.348
Estimated creatinine clearance [ml/min] mean (SD)	38.8 (7.8)	39.0 (8.2)	0.733
Diagnosed hypertension [N] (%)	97 (49.0)	96 (46.6)	0.775
Prescriptions per patient mean (SD)	6.7 (3.0)	7.1 (3.1)	0.318
Prescriptions per patient requiring dose reduction mean (SD)	1.4 (0.9)	1.5 (1.1)	0.124

SD = standard deviation.

## Clinical outcomes

### Patients with potentially dangerous or contraindicated medications

At baseline we registered 2,784 prescriptions of which 588 (21%) required special consideration in CKD. Of these, 270 (46%) were for angiotensin converting-enzyme inhibitors or angiotensin receptor blockers, 77 (13%) for allopurinol, 61 (10%) for antibiotics such as ciprofloxacin, amoxicillin, clavulanic acid, cephalosporins, and 15 (3%) for metformin. There was no significant change in the number of prescriptions overall and per patient at 6 months.

Table 3 displays the number of patients after 6 months receiving medications with a high potential for adverse drug reactions in renal impairment. At baseline, 1 patient had a prescription for nitrofurantoin and 4 patients for metformin in the intervention group versus 11 in the control group. 22/40 patients with a prescription for allopurinol in the intervention group (55%) and 17/37 in the control group (46%) exceeded the recommended standard daily dose. At 6 months, this had been reduced by 18% in the intervention group compared with 1% in the control group. None of the between-group differences at 6 months were statistically significant.

**Table 3:** Patients with potentially dangerous or contraindicated medications at 6 months by intervention and control group

**Variable**	**Intervention group (N = 198 patients)**	**Control group (N = 206 patients)**
Metformin, proportion (%)	6/198 (3.0)	9/206 (4.4)
Nitrofurantoin, proportion (%)	0/198 (0.0)	0/206 (0.0)
Allopurinol, proportion (%)	38/198 (19.2)	40/206 (19.4)
Allopurinol exceeding recommended standard dose, proportion (%)	14/38 (36.8)	18/40 (45.0)

### Patients with prescriptions exceeding the recommended daily dose

The proportion of patients receiving one or more prescriptions exceeding the recommended maximum daily dose was significantly lower in the intervention group compared to the control group after 6 months (19.2% vs. 34.5%; OR = 0.45, 95% CI: 0.29 to 0.70; p < 0.001). This intervention effect remained statistically significant even after adjustment for the proportion of patients with at least one prescription exceeding the recommended maximum daily dose at baseline and further baseline covariates such as patient’s age, gender, underlying diagnosis, and creatinine value (Table 4). The proportion of intervention recipients receiving one or more prescriptions exceeding the recommended standard daily dose by more than 30% was lower than in the control group (47.5% vs. 58.7%; OR = 0.64, 95% CI: 0.41 to 0.98; p = 0.040). However, this difference was not significant after adjustment for baseline covariates (Table 4). A sensitivity analysis of only complete cases revealed no difference in results (data not shown). Because the sample size calculation was based on the estimated effect size of the primary outcome, we did not perform any statistical analyses for the overall number of prescriptions shown above.

**Table 4:** Patients with prescriptions exceeding the recommended daily dose at 6 months by intervention and control group

**Variable**	**Intervention group (N = 198)**	**Control group (N = 206)**	**Estimated model adjusted ICC**	**Adjusted OR (95% CI)**	**p-value**
Patients with ≥1 prescription exceeding recommended maximum dose [N] (%)	38 (19.2)	71 (34.5)	0.001	0.46 (0.26;0.82)	0.008
Patients with ≥1 prescription exceeding recommended standard dose by >30% [N] (%)	94 (47.5)	121 (58.7)	0.039	0.66 (0.36;1.21)	0.180

Adjusted for underlying diagnosis, age, gender, serum creatinine, N patients with ≥1 prescription exceeding recommended maximum or standard dose at baseline.

## Discussion

Our intervention reduced the proportion of patients with prescriptions exceeding the recommended maximum daily dose, with a trend in reducing the proportion of patients with at least one prescription exceeding the recommended standard daily dose by more than 30%.

Most prescriptions exceeding the maximum or standard daily dose were ACE-inhibitors (ACEI) and angiotensin-receptor blockers (ARB).

A sensitivity analysis excluding these drugs failed to show a significant reduction of the recommended daily maximum dose at 6 months (data not shown). This suggests that the intervention effect was mostly related to ACEI and ARB.

Studies show that treatment with ACEI and ARB reduces the progression of CKD [9], e.g. Benazepril was associated with a 52% reduction in the level of proteinuria and a 23% reduction in the rate of decline in renal function [31]. While lower doses may be sufficient to treat hypertension [32,33], dose escalation particularly in CKD may be beneficial due to a nephroprotective effect [31,34]. Although ACEI and ARB have a wide therapeutic margin, high doses bear a risk of hyperkalemia, hypotension, and acute deterioration of renal function [35]. Therefore, special attention is required when starting these drugs. In order to account for this complex situation, the DOSING programme provided information on ACEI/ARB dosage in a text format, including the specific recommendations for patients with CKD or hypertension. At the same time it allowed the calculation of an individual dose reduction for patients with hypertension.

Our results suggest that physicians using the DOSING programme reduced the dose below the maximum but kept it above the standard daily dose recommended for patients. A further sensitivity analysis showed that this was unrelated to the primary diagnosis of the patient (CKD or hypertension; data not shown). A possible explanation for this could be that the information provided by the programme was too complex and physicians were not certain which patients required a dose reduction.

In CKD patients, metformin may cause life-threatening lactic acidosis [36], nitrofurantoin peripheral neuritis [37], and a standard daily dose of 300 mg allopurinol may result in a hypersensitivity syndrome [38]. No patient had been prescribed nitrofurantoin and fewer patients than in other studies [39] were prescribed excessive metformin doses and, thus, differences between intervention and control group were not significant. However, the intervention group showed an 18% reduction in high dose allopurinol prescriptions.

These results suggest that DOSING may be effective in correcting dosing errors that are due to lack of knowledge or awareness (e.g. of serious adverse effects) and for which the evidence provided is unambiguous, but further studies with larger sample sizes are necessary to confirm these findings.

Most previously published evidence on effective interventions to improve correct dosage adjustment in CKD patients comes from inpatient settings [20,21,23], even though dosage-related medication errors are equally relevant in outpatient primary care settings [40]. Clinical decision support systems (CDSS) have been successful in reducing medication errors and adverse drug events in inpatient settings [41-44]. In primary care, CDSS has also shown benefits, but variability among the types and methods of implementation and inconsistent use was noted [45]. Rapid and easy access to the requested information [46,47] and patient-specific advice [48] are important prerequisites for the use of CDSS in busy practices. The strength of the DOSING programme is that it provides patient-specific prescribing advice at the point of care, which has the potential to improve attention and user response [48].

In a concomitant qualitative study reported elsewhere [49], we performed telephone interviews with family physicians in the intervention group in order to assess usability and the content of the programme. Participating physicians found the content of DOSING generally helpful and informative, and reported that its use improved their awareness of patients with impaired renal function.

As a CD version, DOSING proved feasible and effective in small, not fully computerized family practices; yet the intervention effect may be larger if DOSING is integrated into the EHR with a direct link to the medication and the patient information needed to calculate creatinine clearance.

Feasibility and effectiveness of the intervention may be improved if medication checks, implemented as a continuous background process, would create alerts when appropriate during the prescription of drugs. However, most available commercial EHR systems currently lack the functionalities to provide decision support [50].

There are several possible limitations of the study.

First, selection bias, because the participating practices might have been especially motivated to improve prescribing in CKD patients, therefore introducing a study effect in control practices. In Hesse, physicians are required to participate in pharmacotherapy quality circles regularly. The infrequent prescription of potentially dangerous medications suggests that both groups were conscious of their prescribing behaviour. Therefore, the demonstrated effect may underestimate the real potential of the intervention in less well trained primary care practices.

Second, an intervention period of 6 months is relatively short. Further studies are needed to evaluate the long-term effects of the intervention.

Third, our study did not evaluate clinical outcomes for patients such as adverse effects of drugs requiring dose reduction, and we did not assess confounding factors such as the patients’ compliance to drug therapy. However, our study aimed to assess whether the use of a software tool for the estimation of renal function and the provision of information on corresponding dosage adjustment is feasible in busy small primary care practices, and whether it can effectively change prescription behaviour of family physicians for patients with impaired renal function. In order to measure these effects, we selected process rather than outcome parameters, such as the number of inappropriate prescriptions for these patients. Future studies should assess the effect of the intervention on patient-related clinical outcomes.

Finally, unlicensed use of metformin in patients with mild renal impairment is currently subject to debate [51] making automated decision support difficult without considering the particularities of the individual case. In these situations our tool provided both, information on the labelled contraindication as well as guidance for dose adjustment to minimize the risk of accumulation.

Similarly, strict dose reductions of ACEI and ARB by an automated decision support system will only be appropriate if relevant patient characteristics such as the indication (hypertension vs. renal protection in CKD) are available and considered.

## Conclusions

In conclusion, a multifaceted intervention using a software programme for the estimation of creatinine clearance and subsequent dose adjustment is effective in detecting CKD in at-risk patients and reducing inappropriately high doses of renally excreted medications in patients with CKD in small primary care practices. Further research should assess additional costs and benefits if decision support is fully integrated in the EHR.

## Competing interests

The authors declare that they have no competing interests.

## Authors’ contributions

AE, MB, KS, TR and WEH developed intervention and study protocol. FMG and JJP contributed to the development of the study protocol. JR participated in the design of the study and performed the statistical analysis. AE wrote the first draft of the manuscript. JJP and MB critically revised it. All authors read and approved the final manuscript.

## Pre-publication history

The pre-publication history for this paper can be accessed here:

http://www.biomedcentral.com/1471-2296/13/91/prepub
